# Supplementary material for: Modeling glioblastoma heterogeneity as a dynamic network of cell states
Source: Mol Syst Biol. 2021 Sep 16;17(9):e10105. doi: 10.15252/msb.202010105 (PMC8444284; doi:10.15252/msb.202010105)
Supplement: Supplementary file 6 — Source Data for Figure 5 [file MSB-17-e10105-s004.zip › Figure5A_sourcedata/GSEA_3017/hallmarks_stateC.GseaPreranked.1621934520804/HALLMARK_E2F_TARGETS.html]

Details for gene set HALLMARK\_E2F\_TARGETS[GSEA]

|  || Dataset | state13017 |
| Phenotype | NoPhenotypeAvailable |
| Upregulated in class | na\_pos |
| GeneSet | HALLMARK\_E2F\_TARGETS |
| Enrichment Score (ES) | 0.7027637 |
| Normalized Enrichment Score (NES) | 4.462161 |
| Nominal p-value | 0.0 |
| FDR q-value | 0.0 |
| FWER p-Value | 0.0 |
Table: GSEA Results Summary

  

Fig 1: Enrichment plot: HALLMARK\_E2F\_TARGETS      
 Profile of the Running ES Score & Positions of GeneSet Members on the Rank Ordered List

  

| PROBE | GENE SYMBOL | GENE\_TITLE | RANK IN GENE LIST | RANK METRIC SCORE | RUNNING ES | CORE ENRICHMENT || 1 | ATAD2 |  |  | 7 | 0.649 | 0.0310 | Yes |
| 2 | EZH2 |  |  | 14 | 0.574 | 0.0588 | Yes |
| 3 | RRM2 |  |  | 19 | 0.542 | 0.0880 | Yes |
| 4 | HMGB2 |  |  | 20 | 0.534 | 0.1239 | Yes |
| 5 | UBE2T |  |  | 31 | 0.475 | 0.1378 | Yes |
| 6 | ORC6 |  |  | 39 | 0.450 | 0.1554 | Yes |
| 7 | MMS22L |  |  | 40 | 0.448 | 0.1855 | Yes |
| 8 | RAD51AP1 |  |  | 46 | 0.435 | 0.2057 | Yes |
| 9 | PCNA |  |  | 48 | 0.435 | 0.2331 | Yes |
| 10 | TMPO |  |  | 49 | 0.432 | 0.2622 | Yes |
| 11 | TOP2A |  |  | 52 | 0.426 | 0.2872 | Yes |
| 12 | SPC25 |  |  | 53 | 0.415 | 0.3150 | Yes |
| 13 | BRCA1 |  |  | 54 | 0.410 | 0.3425 | Yes |
| 14 | PSIP1 |  |  | 73 | 0.374 | 0.3354 | Yes |
| 15 | DEK |  |  | 74 | 0.373 | 0.3604 | Yes |
| 16 | NASP |  |  | 75 | 0.371 | 0.3853 | Yes |
| 17 | POLD3 |  |  | 76 | 0.367 | 0.4100 | Yes |
| 18 | SMC4 |  |  | 78 | 0.365 | 0.4327 | Yes |
| 19 | STMN1 |  |  | 85 | 0.357 | 0.4459 | Yes |
| 20 | BARD1 |  |  | 86 | 0.357 | 0.4699 | Yes |
| 21 | DUT |  |  | 87 | 0.356 | 0.4938 | Yes |
| 22 | MCM7 |  |  | 91 | 0.352 | 0.5121 | Yes |
| 23 | USP1 |  |  | 92 | 0.352 | 0.5357 | Yes |
| 24 | SLBP |  |  | 106 | 0.331 | 0.5346 | Yes |
| 25 | GINS4 |  |  | 112 | 0.325 | 0.5475 | Yes |
| 26 | MCM3 |  |  | 114 | 0.324 | 0.5674 | Yes |
| 27 | PSMC3IP |  |  | 118 | 0.319 | 0.5835 | Yes |
| 28 | TIMELESS |  |  | 119 | 0.319 | 0.6049 | Yes |
| 29 | SMC1A |  |  | 125 | 0.312 | 0.6169 | Yes |
| 30 | DNMT1 |  |  | 127 | 0.311 | 0.6360 | Yes |
| 31 | LMNB1 |  |  | 142 | 0.293 | 0.6305 | Yes |
| 32 | ASF1B |  |  | 143 | 0.291 | 0.6501 | Yes |
| 33 | RFC3 |  |  | 156 | 0.283 | 0.6475 | Yes |
| 34 | MYBL2 |  |  | 164 | 0.278 | 0.6536 | Yes |
| 35 | HELLS |  |  | 171 | 0.271 | 0.6611 | Yes |
| 36 | MAD2L1 |  |  | 174 | 0.270 | 0.6756 | Yes |
| 37 | MSH2 |  |  | 180 | 0.265 | 0.6844 | Yes |
| 38 | MCM4 |  |  | 189 | 0.259 | 0.6874 | Yes |
| 39 | CDK1 |  |  | 191 | 0.255 | 0.7028 | Yes |
| 40 | MYC |  |  | 421 | -0.336 | 0.3142 | No |
Table: GSEA details [plain text format]

  

Fig 2: HALLMARK\_E2F\_TARGETS: Random ES distribution      
 Gene set null distribution of ES for **HALLMARK\_E2F\_TARGETS**
